# Use of Wearable Sensors to Assess the Effects of Performing a Cognitive Task on Sensory Integration of Balance in Healthy Individuals

**DOI:** 10.3390/s22072776

**Published:** 2022-04-05

**Authors:** Emily Tweel, Arnold J. Stromberg, Geetanjali Gera

**Affiliations:** 1 Human Health Sciences, College of Health Sciences, University of Kentucky, Lexington, KY 40506, USA; evtw222@g.uky.edu; 2 Department of Statistics, University of Kentucky, Lexington, KY 40506, USA; stromberg@uky.edu; 3 Department of Physical Therapy, College of Health Sciences, University of Kentucky, Lexington, KY 40506, USA

**Keywords:** balance, postural sway, sensors, cognition, dual task, sensory–motor interaction, Clinical Test of Sensory Interaction of Balance (mCTSIB)

## Abstract

This study investigated the effects of performing a cognitive task on the sensory integration of balance in healthy individuals. Ten subjects (five F/five M; 21.5 ± 2.17 years; 69.9 ± 3.4 inches; 155.6 ± 26.1 lbs; Caucasian), without known balance issues, performed the modified Clinical Test of Sensory Interaction of Balance (mCTSIB) with and without a cognitive task. The cognitive task involved counting down in threes from a randomly assigned number between 95 and 100. Postural sway area and postural sway jerk were assessed through the use of inertial sensors placed around the subjects’ lower lumbar region. Each subject performed four trials for the four conditions of the mCTSIB: eyes open firm (EOFirm), eyes closed firm (ECFirm), eyes open foam (EOFoam), and eyes closed foam (ECFoam). We tested the effect of performing a cognitive task on the sensory integration of balance. We hypothesized that sensory cognitive interaction would be more apparent for more complex conditions and would be better assessed with postural sway jerk compared to postural sway area measure. With the addition of a cognitive task for the mCTSIB: (1) postural sway area increased in the baseline condition, i.e., EOFirm (*p* < 0.05), but did not increase in the most difficult condition, i.e., ECFoam; (2) postural sway jerk increased in all conditions of the mCTSIB (*p* < 0.05); (3) cognitive performance did not deteriorate across conditions of the mCTSIB. Postural sway jerk was shown to be a more sensitive measure in detecting the effect of a cognitive task on sensory integration for postural control. Overall, inertial sensors can be used to reliably assess postural sway differences related to sensory–cognitive integration.

## 1. Introduction

Dual-tasking, i.e., performing a motor task with a cognitive task, is commonly performed in day to day activities [1]. Dual-tasking often requires integrating sensory inputs, i.e., visual, vestibular, and somatosensory information, with a postural (balance) task while performing a cognitive task. The ability to perform postural and cognitive tasks simultaneously becomes compromised with various neurological and neurodegenerative diseases. [2,3,4,5]. Thus, it is important to be able to quantify the interaction of sensory integration during a postural task while simultaneously performing a cognitive task [4,6].

The integration of information from the three sensory systems, i.e., somatosensory, visual, and vestibular, helps maintain human upright balance/postural control [7]. The somatosensory system carries information to determine the body’s position related to itself [7]. The visual system provides input to determine the body’s relationship with other objects [7], whereas the vestibular system helps to detect the position of the body with respect to the earth [8]. Individual contributions from the three sensory systems for postural control, i.e., sensory reweighting, varies depending on the context [7].

The sensory organization test (SOT) is the clinical gold standard for assessing sensory-integration deficits. SOT uses sway referencing force plates and a visual surround on an Equitest (NeuroCom, Clackamas, Oregon) balance device to assess balance. Although the SOT is the gold standard test, the equipment is expensive, has a large footprint, and is not portable. A less expensive and portable substitution for the SOT is the modified Clinical Test of Sensory Interaction of Balance (mCTSIB) [9]. This stopwatch-based test assesses a person’s balance in quiet stance while standing on a firm or foam surface with their eyes open or closed. The quantification of postural sway with the inertial sensors can provide objective measures that are more sensitive in detecting balance deficits than the stop-watch-based clinical measure of mCTSIB [10,11]. Body-worn inertial sensors have been validated with force platforms to produce reliable and automatic measures of postural sway [11,12].

Cognitive–postural interference could cause deterioration in either cognitive or postural task performance when individuals perform cognitive and postural tasks simultaneously [3,6,13,14,15]. Cognitive–postural interference could cause a person to lose their balance or be unable to perform the cognitive task [13]. Thus, it is important to assess sensory integration during cognitive task performance [16,17,18,19,20,21,22,23,24]. To our knowledge, our study is the first to assess the interaction of cognition and sensory integration using the instrumented mCTSIB in healthy individuals. We hypothesized that the addition of a cognitive task (dual task (cog) versus single task (non-cog)) would lead to an increase in postural sway, especially for the more complex conditions. Furthermore, we hypothesized that these differences in the cognitive and non-cognitive tasks would be more evident with sensitive measures based of the inertial sensors. We also assessed the test–retest reliability of the instrumented mCTSIB (cog and non-cog).

## 2. Materials and Methods

### 2.1. Participants

Eleven healthy, college-aged students were recruited to perform the instrumented mCTSIB. Data for one individual could not be used due to technical issues. Study participants did not report any balance problems or cognitive issues. The ten individuals included five females and five males (age: 21.5 ± 2.17 years; height: 69.9 ± 3.4 inches; weight: 155.6 ± 26.1 lbs), and all were Caucasian in race. All subjects signed an informed consent form approved by the Institutional Review Board of the University of Kentucky.

### 2.2. Procedure

**Figure 1:**
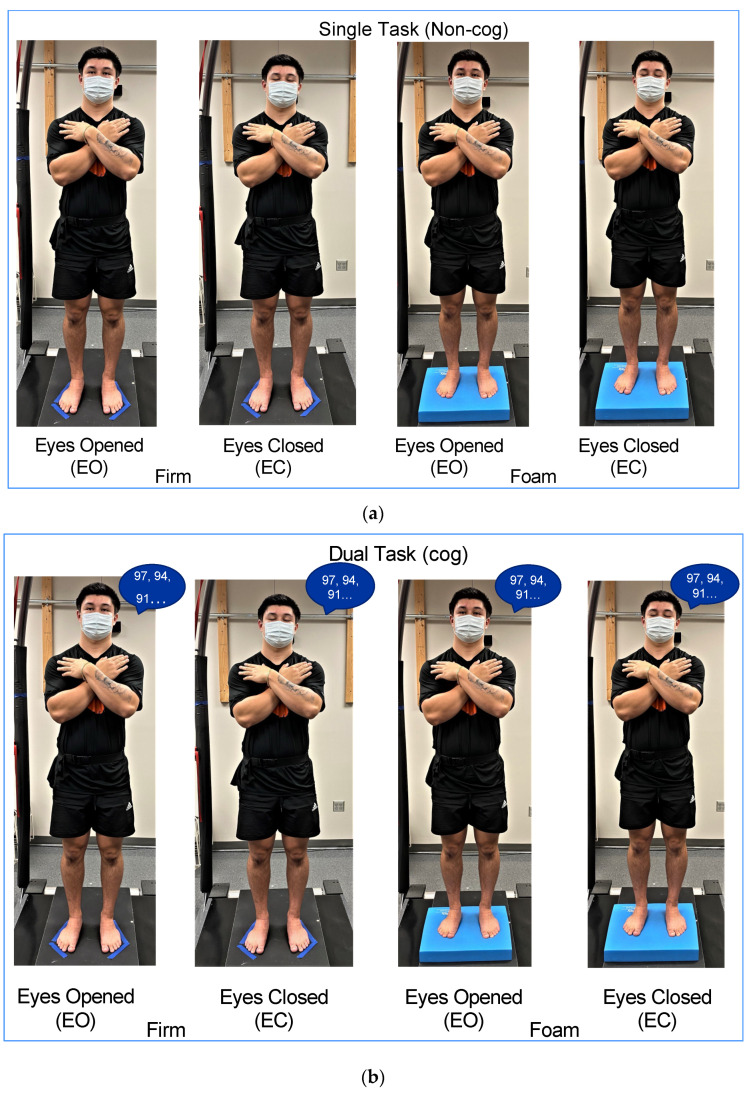
(**a**). Four conditions for the mCTSIB without a cognitive task (*non-cog mCTSIB*). (**b**). Four conditions for the mCTSIB with a cognitive task (*cog mCTSIB*).

Subjects performed mCTSIB without (single task: *non-cog mCTSIB*; Figure 1a) and with a cognitive task (dual task: *cog mCTSIB*; Figure 1b) to test the interaction of the sensory integration of balance and cognition. The mCTSIB includes four conditions with individuals standing with their: (1) *eyes open* on *firm surface* (EOFirm), (2) *eyes closed* on *firm surface* (ECFirm), (3) *eyes open* on *foam surface* (EOFoam), and (4) *eyes closed* on *foam surface* (ECFoam). Subjects performed four trials for each condition, with each trial lasting for 30 s. The foam pad was flipped over between EOFoam and ECFoam conditions. Subjects had a two-minute break and were required to remain standing between the non-cog and cog mCTSIB. The order effect was controlled by alternating the testing of the *non-cog mCTSIB* and *cog mCTSIB* between odd- and even-numbered subjects.

For the *cog mCTSIB*, a random number between 95 and 100 was chosen, and subjects were asked to count backwards from that number by three. A different starting number was selected across trials to reduce memorization confounds during the cognitive task. Cognitive performance was assessed by recording the correct numbers recited by individuals for each trial. Errors were not included in the number count, but self-corrections were included.

Subjects stood barefoot with their arms crossed over their chest. Subjects were instructed to look straight ahead at an “X” that was taped to the wall 6ft. distance from them at their eye height. Stance width was controlled by having subjects stand at a standardized feet template width apart (APDM, Inc., Portland, OR, USA). Subjects’ feet outline was marked to ensure a consistent initial position across trials. Postural sway was assessed using the inertial sensor (Opal; APDM, Inc., Portland, OR, USA), placed around the waist close to the lumbar fourth to fifth vertebra with an elastic strap. The inertial sensor was composed of a tri-axial accelerometer, a tri-axial gyroscope, and a tri-axial magnetometer. Postural sway was assessed based on the acceleration signals from the inertial sensor [12].

The test–retest reliability of the *non-cog mCTSIB* and *cog mCTSIB* was assessed by having subjects repeat the experimental paradigm after a 30-min break. The sequence of the mCTSIB was kept intact, as the traditional clinical test, i.e., EOFirm was performed first, followed by ECFirm, EOFoam, and ECFoam. The order of non-cog and cog mCTSIB for the retest session was also kept consistent with the test session.

### 2.3. Outcome Measures

We used the total postural sway area and postural sway jerk, automatically calculated with Mobility lab V1 software (APDM, Inc., Portland, OR), as the primary outcomes of postural sway [12]. The postural sway area was computed by the area spanned by the acceleration signal per unit of time. Postural sway jerk was the time derivative of the acceleration signal [12]. These postural sway measures were compared across conditions for the *non-cog mCTSIB* and between the *non-cog mCTSIB* and *cog mCTSIB*.

### 2.4. Statistical Analysis

**Figure 2:**
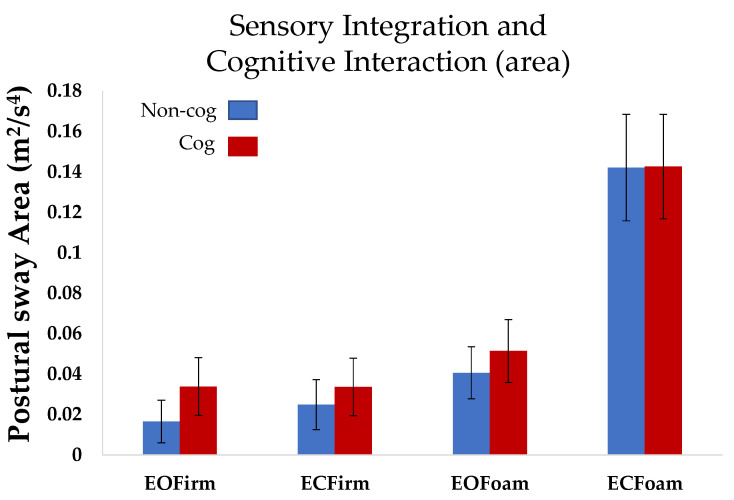
Mean postural sway area across all subjects for the *non-cog mCTSIB* and *cog mCTSIB*. Error bars represent standard error of the mean. *Non-cog* (mCSTIB without cognitive task); *cog* (mCSTIB with cognitive task).

**Figure 3:**
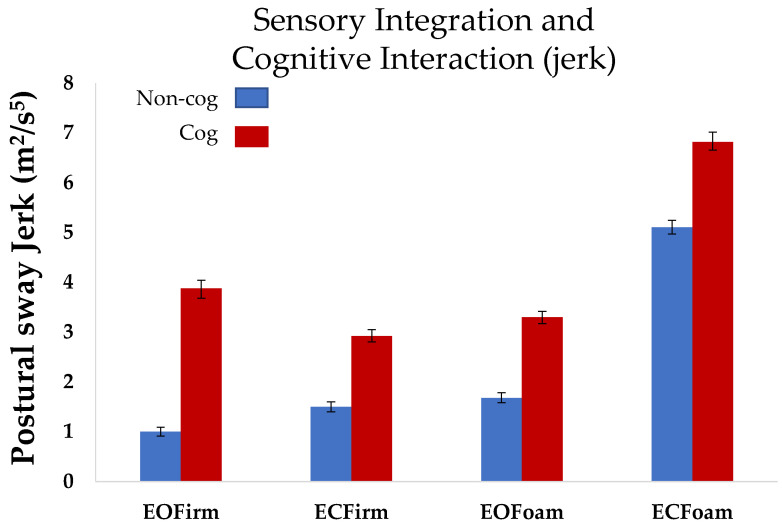
Mean postural sway jerk across all subjects for the *non-cog mCTSIB* and *cog mCTSIB*. Error bars represent the standard error of the mean. *Non-cog* (mCSTIB without cognitive task); *cog* (mCSTIB with cognitive task).

Statistical analyses were performed using the JMP software 16.0. The normality of data distribution was assumed. Multiple comparisons were made using Tukey’s honest significant difference. Differences were assumed significant when the *p*-value was less than 0.05. One-way ANOVA with repeated measures, using compound symmetry, was used to assess postural sway across conditions (*non-cog mCTSIB*) (Figure 2). Two-way ANOVA with repeated measures, using compound symmetry, was used to assess the interaction between sensory integration and cognition (the *non-cog mCTSIB* versus *cog mCTSIB*), (Figure 3).

## 3. Results

Postural sway measures did not differ for the test–retest sessions for the non-cog mCTSIB and cog mCTSIB (*p* = 0.87). Therefore, for the subsequent analyses, data were averaged for the test and retest sessions.

### 3.1. Sensory Integration during mCTSIB (Non-Cog)

*Postural sway area:* When the mCTSIB was performed without a cognitive task (the *non-cog mCTSIB*), we observed an increase in the postural sway area as the task complexity increased. The postural sway area in the most difficult condition (ECFoam) was significantly higher than that in the other three conditions (EOFirm (*p* < 0.0001), ECFirm (*p* < 0.0001), and ECFoam (*p* < 0.0001); overall comparison: F_3,27_ = 39.6, *p* < 0.0001, Figure 2).

*Postural sway jerk:* Similar to the postural sway area, we observed an increase in the postural sway jerk as the task complexity increased during the *non-cog mCTSIB*. Postural sway jerk in the most difficult condition (ECFoam) was significantly higher that in the other three conditions (EOFirm (*p* < 0.0001), ECFirm (*p* < 0.0001), and ECFoam (*p* < 0.0001); overall comparison: F_3,27_ = 63.6, *p* < 0.0001, Figure 3).

### 3.2. Sensory Integration and Cognitive Interaction (mCTSIB: Non-Cog vs. Cog)

**Postural sway area:** Overall, repeated measures ANOVA did not reveal an interaction effect of cognition on sway area for the four conditions of mCTSIB (*p* = 0.82). However, individual comparison for the conditions revealed that sway area for the baseline condition (EOFirm), was higher for the cognitive task compared to the non-cognitive task, i.e., EOFirmnon-cog < EOFirmcog (*p* < 0.005, Figure 2).

**Postural sway jerk:** Repeated measures ANOVA revealed a main effect of cognition in the postural sway jerk measure (*p* < 0.001). The effect of cognition on jerk was similar in the four conditions of mCTSIB (*p* = 0.55). Thus, postural sway jerk in the cognitive task (cog mCTSIB), was significantly higher than that in the non-cognitive task (non-cog mCTSIB) for all conditions (*p* < 0.001, Figure 3).

### 3.3. Cognitive Performance

Table 1 shows the cognitive performance, i.e., the total numbers recited averaged across four trials for individual conditions of cog mCTSIB. Cognitive performance did not deteriorate as the task complexity of the mCTSIB increased (Table 1, F_3,36_ = 0.56, *p* = 0.64; all comparisons: *p* > 0.28). Overall, subjects did not exhibit deficits in the cognitive task performance. The range of cognitive errors for subjects across conditions was between 0 and 3.

### 3.4. Correlation of Postural Sway Measures across Subjects

**Figure 4:**
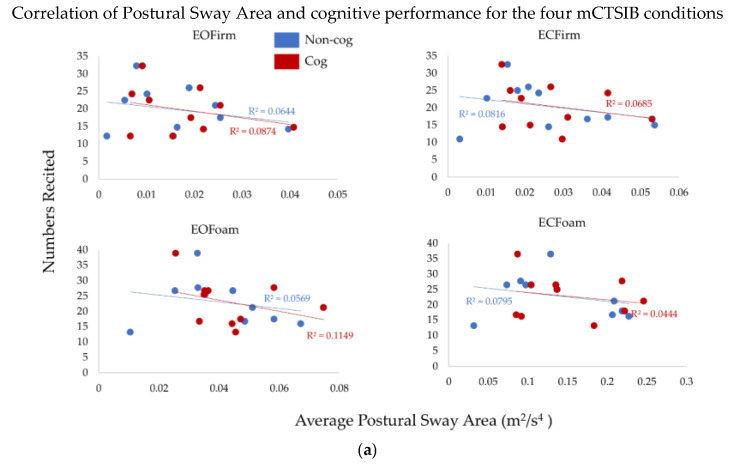

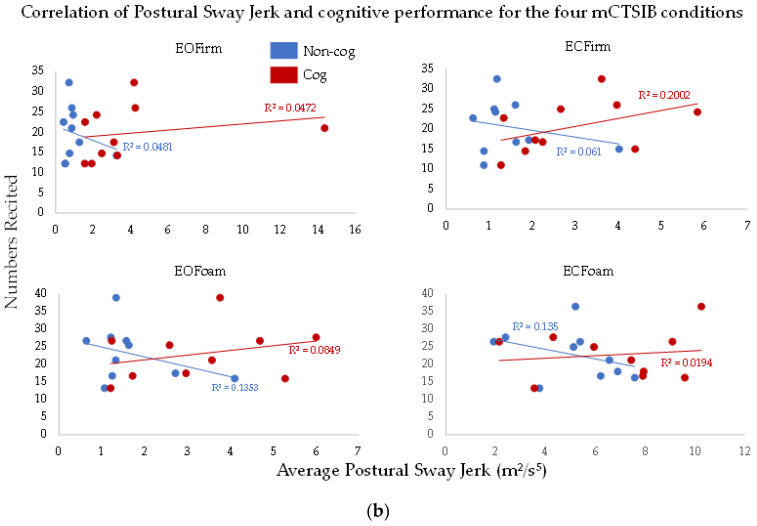
The correlation of (**a**) postural sway area and (**b**) postural sway jerk with cognitive performance, i.e., total correct numbers recited for the conditions of *cog mCSTIB*. Non-cog is the *non-cog mCTSIB* (mCSTIB without cognitive task), and cog is the *cog mCTSIB* (mCSTIB with cognitive task).

To understand the effects of the complexity of sensory integration on the cognitive task (the total numbers recited), we performed correlation analysis between postural sway measures and cognitive performance (Figure 4a,b). There was no significant relationship between postural sway measures and cognitive performance (*p* > 0.7, R^2^ > 0.01, for all comparisons). Thus, the total numbers recited did not relate to the postural sway measures. Furthermore, we did not observe this relationship to change across conditions of the cog mCTSIB.

### 3.5. Postural Sway Measures for Two Exemplar Subjects

We assessed two subjects at different ends of the spectrum for cognitive performance, i.e., the highest and lowest number of numbers recited for the cognitive task. Subject A recited the maximum number of numbers, whereas subject B recited the minimum number of numbers during the cognitive task. Irrespective of the total numbers recited by both individuals, cognitive performance did not show deterioration across the four conditions of the mCTSIB (Table 1). Additionally, the cognitive errors for both subjects ranged between 0 and 1.

As shown in Table 2, consistent with the group average, Subjects A and B showed an increase in postural sway area as the task complexity increased for the non-cog mCTSIB. However, Subjects A and B showed different postural sway areas from the group average for sensory integration in the cog mCTSIB. Subject A displayed an increase in postural sway area as well as postural sway jerk with the increasing task complexity of the mCTSIB. This increase was more evident for the jerk measure as compared to the area measure. However, subject B showed an increase in postural sway area with the addition of a cognitive task only in the baseline condition (EOFirm), whereas postural sway jerk increased in most of the conditions of the mCTSIB, except for ECFoam.

## 4. Discussion

In this study, we investigated the interaction of performing a cognitive task with sensory integration for balance in healthy individuals. Our primary findings are: (1) with the addition of a cognitive task for the mCTSIB (*cog mCTSIB*), postural sway area increased in the baseline condition, i.e., EOFirm, but did not increase in the most difficult condition, i.e., ECFoam; (2) postural sway jerk increased in all conditions of the mCTSIB with the addition of a cognitive task (*cog mCTSIB*); (3) cognitive performance did not deteriorate across conditions of the mCTSIB. These findings were consistent when we retested our subjects after a 30-min break the same day.

### 4.1. Postural Sway Changes with Sensory Integration and Cognitive Interaction

Postural sway area increased as the task complexity increased across the sensory conditions for the *non-cog mCTSIB*. The effects of performing a cognitive condition on the mCTSIB were observed as an increase in postural sway area in the baseline condition (EOFirm). However, in the most difficult condition of mCTSIB (ECFoam), we did not observe any change in postural sway area with the addition of a cognitive task. Differences in the sensory cognitive interaction across conditions could be attributed to the achievement of limits of stability during the non-cognitive ECFoam and not having any further range to increase postural sway during the cognitive ECFoam.

### 4.2. Postural Sway Jerk: A Measure to Assess Optimization of Postural and Cognitive Performance

The ability to maintain postural sway area for ECFoam while performing the cognitive task could be explained by the increased higher-order adjustments (jerk) of the postural sway. The subjects potentially reached their limits of stability for the most challenging condition, i.e., ECFoam, and maintained their balance by quick adjustments of postural sway, as is evident by increased jerk for ECFoam. Thus, postural sway jerk provides a window to understand subtle changes involved in sensory integration and cognitive interaction. It is conceivable that the inconsistencies across the existing literature related to interaction of sensory integration and cognitive task could be clarified with the use of additional sensitive measures such as postural sway jerk [12,25]. We also observed preserved cognitive performance across the increasing task complexity of mCTSIB. Interestingly, cognitive errors were also relatively low for our cohort.

### 4.3. Heterogeneity for Sensory Cognitive Integration across Existing Literature

The effects of performing a cognitive task with the sensory integration of balance lacks consensus across the literature, with some studies observing a reduction while others an increase or unchanged postural performance with the addition of a cognitive task [16,17,18,19,20,21,22,23,24]. Table 3 provides a summary of studies that assessed the interaction of cognitive and sensory integration, where the latter was tested based on the SOT and mCTSIB [16,17,18,22,23]. The SOT uses sway referencing force plates and visual surrounds on the Equitest (NeuroCom, Clackamas, OR, USA) balance device to assess sensory integration with six test conditions (C1:C6).

As seen in Table 3, for the baseline condition, most studies noticed a reduction in postural performance with the addition of a cognitive task. Inconsistencies across studies are more evident when the demand for sensory integration complexity increases, e.g., ECFoam of the mCTSIB or C6 of the SOT (Table 3). The heterogeneity of the study reports could be attributed to differences across postural measures used to assess balance. The difficulty level of each cognitive task was also variable across these studies. Most of the studies found that cognitive performance decreased as the task complexity increased [16,17,18,20,21], and some did not report cognitive performance while dual-tasking [19,22,23,24].

### 4.4. Sensory Cognitive Interaction: A Reflection of Optimization and Strategy Selection

The effects of sensory cognitive interaction could depend on the strategy used while completing the balance and cognitive task concurrently, i.e., prioritizing cognitive versus postural control [16,17,18,19,20,21,22,23,24]. For example, Resch et al., found that when performing a dual task (balance task plus cognitive task), participants appeared to prioritize postural control over cognitive processing, with cognitive performance getting more affected with the increasing demands of postural task [17]. This finding suggests that dual-tasking could result in less change in postural control but slower cognitive processing. Our subjects were likely able to perform cognitive tasks without the deterioration of cognitive performance because of the simplicity of the cognitive task (Table 1). Although we did not observe any change in postural sway area for the most difficult condition (Figure 2), there were subtle postural sway adjustments to incorporate the interaction of cognition with postural control demands. These adjustments were evident by the increase in postural sway jerk consistently across all cognitive conditions (Figure 3).

### 4.5. Comparison of Postural Sway Measures for Two Example Subjects with Maximum and Minimum Numbers Recited for Cognitive Task

We did observe variability across subjects in the total numbers recited for the cognitive task. However, despite this variability (as shown in Table 1 and Table 2 for our two subjects with maximum and minimum numbers of numbers recited), we did not observe a deterioration in the cognitive performance across the increasing difficulty of task complexity for the mCTSIB. This was also accompanied with a similar range of cognitive errors across subjects.

We did not find any significant association between the total numbers recited and postural sway measures (Figure 4). However, the results for two exemplar subjects with maximum and minimum numbers recited are interesting and contrasting with each other. Our Subject A, who recited the maximum number of numbers, did show an increase in both postural sway measures with the addition of a cognitive task (Table 2), whereas the subject who recited the minimum number of numbers, subject B, showed an increase in postural sway area only for the baseline condition. Interestingly, for subject B, like our group results, the postural sway jerk measure was more sensitive in detecting the interaction of cognitive task and postural control (Table 2). These findings suggest that individuals who took longer recall time might have used more cautious behavior in maintaining balance, whereby the increase in postural sway measures was less evident.

### 4.6. Effects of Experimental Design on Increase or Decrease in Postural Sway

Inconsistencies across studies, performed on young adults, for the interaction of cognition and postural control could be because of experimental design variability, i.e., instruction to subjects, the difficulty level of cognitive and postural task, and practice of the task [16,17,18,19,20,21,22,23,24]. Most studies involved instructing their subjects to focus on standing still while performing the balance test [16,17,18,19,20,21,22,23,24]. We asked our subjects to stand upright while maintaining their balance and count backwards in threes starting from a given number. Thus, we did not emphasize prioritizing either the cognitive or postural task.

The difficulty level of the cognitive task can cause a greater challenge on sensory integration requirements for postural control. This challenge can be even greater if the demand for the postural task increases simultaneously. Our cognitive task required individuals to recite numbers backward in threes anywhere from a number chosen from 95 to 100. Thus, our cognitive task was relatively simpler compared to some of the other studies [16,17,18,21,22,23]. For example, having subjects solve and announce their responses/solutions to arithmetic equations with multiple functions involved [22] or having individuals count backwards in sevens [23]. *Despite the simplicity of our cognitive task, we did observe effects of cognitive demands on postural control, as is evident by subtle changes observed in postural sway* *jerk.*

The robustness of our findings is also bolstered by the fact that we randomized the order of the performance of cognitive versus non-cognitive task across subjects. We did not observe an order effect, i.e., it did not matter whether the individuals performed the cognitive or the non-cognitive task first. In addition, when we test–retested our individuals for the entire experiment with mCTSIB, our results showed similar postural sway measures. Thus, practice did not change the postural sway performance.

### 4.7. With the Use of Traditional Measures, Baseline Condition Might Be Better to Detect Cognitive Postural Interaction

Although we observed more variability for the postural sway area for ECFoam with and without cognitive tasks, our findings for EOFirm were very consistent across subjects. The baseline condition of eyes open on a firm surface showed an increase in postural sway area with the cognitive task as compared to the non-cognitive task for all subjects. An increase in postural sway area with the cognitive task for EOFirm was evident between our most contrasting individuals as well, i.e., with the lowest and the highest number of correct numbers recited. Thus, based on our data, we suggest that when detecting differences in the cognitive versus non-cognitive tasks, it might be better to assess individuals’ postural sway area in the simplest condition. EOFirm is not affected by the complexity of the task because it does not put an individual to the limits of their postural stability, whereas, to determine the strategy, i.e., cognitive versus postural, ECFoam might be the condition of choice. Thus, if sensitive measures (jerk) with the use of inertial sensors are inaccessible, traditional assessment might consider a baseline condition to test the effects of the interaction of postural and cognitive tasks and more difficult conditions to estimate the prioritization of cognitive versus postural strategies.

## 5. Conclusions

Inertial sensors can reliably be used to assess postural sway differences related to the interaction of sensory integration and cognitive tasks. Postural sway jerk could prove to be a more sensitive measure in detecting the effects of cognitive tasks on sensory integration for postural control.

Limitations: The small sample size, along with the heterogeneity of results across subjects, remains a limitation of our study. A larger sample size study will be able to aid in drawing conclusive results about the interaction of sensory integration and cognition.

## Figures and Tables

**Table 1:** Average (±SD) cognitive performance across all subjects for each condition of *cog mCTSIB*. The two exemplar subjects include individuals with the highest (Subject A) and lowest (Subject B) cognitive performance, i.e., total correct numbers recited for the *cog mCTSIB*.

Cognitive Performance	EOFirm	ECFirm	EOFoam	ECFoam
Average	19.70 ± 1.71	20.50 ± 0.79	23.05 ± 0.96	22.78 ± 1.44
Subject A	32.25	32.50	39.00	36.50
Subject B	12.25	11.00	13.25	13.25

**Table 2:** The postural sway area and postural sway jerk for the two exemplar subjects, with the highest (Subject A) and lowest (Subject B) cognitive performance, i.e., total correct numbers recited for the *cog mCTSIB*.

		Postural Sway Area (m^2^/s^4^)		Postural Sway Jerk (m^2^/s^5^)	
mCTSIB Condition	Cognitive Condition	Subject A	Subject B	Subject A	Subject B
EOFirm	Non-cog	0.008	0.002	0.708	0.496
	Cog	0.031	0.003	4.177	1.913
ECFirm	Non-cog	0.016	0.003	1.179	0.871
	Cog	0.033	0.003	3.609	1.273
EOFoam	Non-cog	0.033	0.011	1.332	1.061
	Cog	0.053	0.005	3.757	1.201
ECFoam	Non-cog	0.129	0.031	5.199	3.754
	Cog	0.220	0.026	10.252	3.548

**Table 3:** Comparison of the existing literature to our study in reference to sensory–cognitive interaction. Most of the studies tested sensory integration of balance using the Sensory Organization Test (SOT) rather than the modified Clinical Test of Sensory Integration of Balance (mCTSIB). Our study (Tweel et al.) utilized instrumented mCTSIB. > sign indicates that the postural performance was worse for the respective comparison, whereas < sign indicates the postural sway measures were better for the respective comparison.

SOT Studies	C1	C2	C3	C4	C5	C6
Lanzarin 2015	Cog>Non-cog	Cog>Non-cog	Cog<Non-cog	Cog>Non-cog	Cog>Non-cog	Cog>Non-cog
Broglio 2005	Cog<Non-cog		Cog<Non-cog	Cog<Non-cog		Cog=Non-cog
Resch 2011	Cog<Non-cog	Cog<Non-cog	Cog<Non-cog	Cog=Non-cog	Cog<Non-cog	Cog=Non-cog
Morelli 2020	Cog>Non-cog	Cog>Non-cog	Cog>Non-cog	Cog=Non-cog	Cog>Non-cog	Cog>Non-cog
mCTSIB Studies	EOFirm	ECFirm	EOFoam	ECFoam		
Ketcham 2018	Cog<Non-cog	Cog<Non-cog	Cog<Non-cog	Cog>Non-cog		
Tweel (current)	Cog>Non-cog	Cog>Non-cog	Cog>Non-cog	Cog=Non-cog

## Data Availability

Data are stored on the Gera lab server, housed in the College of Health Sciences at the University of Kentucky.
